# Enhanced infection control interventions reduced catheter-related bloodstream infections in the neonatal department of Hung Vuong Hospital, Vietnam, 2011–2012: a pre- and post-intervention study

**DOI:** 10.1186/s13756-019-0669-1

**Published:** 2020-01-07

**Authors:** Hang Thi Phan, Thuan Huu Vo, Hang Thi Thuy Tran, Hanh Thi Ngoc Huynh, Hong Thi Thu Nguyen, Truong Van Nguyen

**Affiliations:** 1 Department of Infection Control, Hung Vuong Hospital, 128 Hong Bang Street, District 5, Ho Chi Minh City, Vietnam; 2 Hung Vuong Hospital, 128 Hong Bang Street, District 5, Ho Chi Minh City, Vietnam

**Keywords:** Infection control, Catheter, Bloodstream infection, Intervention, Aseptic technique, Vietnam

## Abstract

**Background:**

Catheter-related bloodstream infections (CR-BSI) cause high neonatal mortality and are related to inadequate aseptic technique during the care and maintenance of a catheter. The incidence of CR-BSI among neonates in Hung Vuong Hospital was higher than that of other neonatal care centres in Vietnam.

**Methods:**

An 18-month pre- and post-intervention study was conducted over three 6-month periods to evaluate the effectiveness of the intervention for CR-BSI and to identify risk factors associated with CR-BSI. During the intervention period, we trained all nurses in the Department of Neonatology on BSI preventive practices, provided auditing and feedback about aseptic technique during catheter care and maintenance, and reorganised preparation of total parenteral nutrition. All neonates with intravenous catheter insertion ≥48 h in the pre- and post-intervention period were enrolled. A standardised questionnaire was used to collect data. Blood samples were collected for cultures. We used Poisson regression to calculate rate ratio (RR) and 95% confidence interval (CI) for CR-BSI incidence rates and logistic regression to identify risk factors associated with CR-BSI.

**Results:**

Of 2225 neonates enrolled, 1027 were enrolled in the pre-intervention period, of which 53 CR-BSI cases occurred in 8399 catheter-days, and 1198 were enrolled in the post-intervention period, of which 32 CR-BSI cases occurred in 8324 catheter-days. Incidence rates of CR-BSI significantly decreased after the intervention (RR = 0.61, 95% CI 0.39–0.94). Days of hospitalisation, episodes of non-catheter–related hospital-acquired infections, and the proportion of deaths significantly decreased after the intervention (*p* < 0.01). The CR-BSI was associated with days of intravenous catheter (odds ratio [OR] = 1.05, 95% CI 1.03–1.08), use of endotracheal intubation (OR = 2.27, 95% CI 1.27–4.06), and intravenous injection (OR = 8.50, 95% CI 1.14–63.4).

**Conclusions:**

The interventions significantly decreased the incidence rate of CR-BSI. Regular refresher training and auditing and feedback about aseptic technique during care and maintenance of catheters are critical to reducing CR-BSI.

## Introduction

Intravenous catheter insertion is vital to managing critically ill patients, but it may involve a high risk of catheter-related bloodstream infections (CR-BSI). Bloodstream infection, usually caused by multi-drug–resistant organisms (MDRO), may be fatal and result in a long hospital stay and high treatment costs [1]. The most common pathogens of CR-BSI are coagulase-negative staphylococci, *Staphylococcus aureus*, and *Enterococcus* spp. [2, 3]. The incidence rates of CR-BSI range from 3.8–11.3 per 1000 neonate-days in neonatal intensive care units in developed countries, accounting for 30% of hospital-acquired infections (HAI) in paediatric hospitals [1, 4, 5]. The rates are usually much higher in developing countries due to hospital overload, insufficient medical equipment, and unsafe aseptic practices by healthcare workers [6]. Guembe et al. showed that the CR-BSI rate in developing countries was 18.7%, while that of the United States was 0.1% [7]. Studies have shown several basic infection control practices such as hand hygiene, skin disinfection, dressing catheter sites, and appropriate intravascular catheters are effective in reducing CR-BSI [8–11].

In Vietnam, CR-BSI rates are relatively high in neonatal intensive care units. At Children’s Hospital No. 1, Ho Chi Minh City in 2005, the CR-BSI rate was 7.5 per 1000 catheter-days, and the BSI from peripheral and central catheters were 6.3 per 1000 patient-days and 15.8% of total admitted cases, respectively [12].

Hung Vuong Hospital (HVH) is one of the largest tertiary maternity hospitals in Vietnam, with 900 beds and more than 40,000 deliveries annually. Department of Neonatology (DON) of the HVH has 100 beds, including 20 in the neonatal intensive care unit (NICU), where about 120 neonates are admitted each month. However, only one doctor and two nurses are available 24/7 in the NICU. It is a heavy workload to take care of 20 severe ill neonates, including preparing total parenteral nutrition (TPN), in NICU. Patient overload and understaffing are high in HVH but most severe in DON.

In 2007, a study conducted in HVH found that the proportion of intravenous catheter utilisations among the total admitted neonates in DON was 34.6%, but the rate of hospital-acquired bloodstream infection (BSI) in the DON was 15.3 per 1000 catheter-days. The proportion of nurses in the DON who complied with aseptic techniques during catheter placement was unacceptably low (25%) [13]. In addition, TPN was prepared in patient rooms, not in the Department of Pharmaceuticals (DOP), as the World Health Organization (WHO) recommends.

At HVH, the high rate of CR-BSI could result from factors related to characteristics of patients, types of catheters, and aseptic techniques of healthcare workers during the placement and maintenance of catheters. Because of limited resources to address all these factors, we intervened in compliance with nurses’ aseptic techniques in DON together with relocating TPN preparation to DOP. The aims of this study were to (i) assess the incidence and causative organisms of CR-BSI, (ii) evaluate the effectiveness of the interventions, and (iii) identify the risk factors associated with CR-BSI.

## Methods

### Descriptive of the intervention

The study was conducted from March 2011 through August 2012 in three succeeding 6-month periods: pre-intervention (01.3.2011–31.8.2011), intervention (01.9.2011–29.02.2012), and post-intervention (01.3.2012–31.8.2012). During the intervention period, we implemented the following: (i) training for all nurses of DON on BSI preventive practices; (ii) auditing of and feedback about aseptic technique during care and maintenance of catheters; and (iii) reorganising the procedure of TPN preparation by preparing TPN under a laminar airflow hood and relocating to a clean room in DOP.

In all, 71 nurses were trained in three one-day hands-on training courses. During the course, intravenous catheter insertion technique was first reviewed. This was followed by a demonstration and practice of handwashing, aseptic technique, maximal sterile barrier protection (e.g. head caps, facemasks, sterile body gowns, and sterile gloves), antiseptic use, skin preparation, and catheter care and maintenance. Training materials for the BSI preventive practices were adapted from the Centers for Disease Control and Prevention (CDC) Guidelines for the Prevention of Intravascular Catheter-Related Infections [1]. All nurses of DON were requested to take a pre- and post-test, and they had to obtain at least 80% of the maximum score on the post-test. The auditing and feedback were recorded and reported in daily meetings in DON to maximise the nurses’ learning, knowledge, and skills.

### Collection of data

Definitions of CR-BSI were slightly adapted from Horan et al. [14], as summarised in Table 1. During the pre- and post-intervention periods, all neonates with intravenous catheters in DON were enrolled in our study. We collected demographic details, health information at birth, time and duration of signs and symptoms of infections, duration of intravenous catheter insertion and hospitalisation, and risk factors of CR-BSI (e.g. gestational age, gender, weight at birth, preterm birth, congenital diseases, methods of delivery, duration of hospitalisation, intravenous injection, blood infusion, parenteral nutrition, endotracheal intubation, duration of ventilator, duration of umbilical intravenous infusion, and HAI) using a standardised questionnaire. We excluded neonates with confirmed BSI at admission or those with intravenous catheter insertion of less than 48 h.

**Table 1:** Definitions of catheter-related bloodstream infections among neonates with intravenous catheter insertion for ≥48 h in Hung Vuong Hospital, Vietnam

Laboratory-confirmed bloodstream infection - A recognized pathogen cultured from blood cultures, and - Organism cultured from blood unrelated to an infection at another site	
Clinical and common skin contaminant bloodstream infection - Fever (> 38 °C, rectal), hypothermia (< 37 °C, rectal), apnea, or bradycardia, and - Signs and symptoms and positive laboratory results were unrelated to an infection at another site, and - Common skin contaminant was cultured from two or more blood	
Clinical bloodstream infection - Fever (> 38 °C, rectal), hypothermia (< 37 °C, rectal), apnea, or bradycardia without evidence of blood culture, and - Unrelated infection at another site, and - Diagnosed sepsis by a clinician of HVH and confirmed CR-BSI after a consultation between clinicians and infection control specialists	

All members of the study team were trained in data collection. Data were processed weekly to ensure completeness and internal consistency.

Blood samples were collected for cultures to detect commonly recognised pathogens (e.g. coagulase-negative staphylococci, *Staphylococcus aureus*, *Enterococcus* spp., *Stenotrophomonas maltophilia*, *Escherichia coli*, and *Acinetobacter spp.*) based on the standard operating procedure of HVH. A multi-drug–resistant (MDR) isolate “is resistant to at least one antibiotic in three or more drug classes” [15]. When antibiotic-resistant bacteria were detected in the HVH laboratories, six antimicrobial classes (aminoglycosides, β-lactam, carbapenems, cephalosporins, fluoroquinolones, and sulbactam) were tested to confirm MDRO based on the Clinical & Laboratory Standards Institute and also referred to laboratory tests approved by the Food and Drug Administration [16].

### Analysis of data

Data were analysed using R software (Epi, BMA, and car packages). Chi-square, Fisher’s exact test, t-test, or Mann Whitney U test was used to compare the characteristics of neonates between the pre- and post-intervention periods with a significance level of < 0.05. Poisson regression was used to calculate rate ratio (RR) and 95% confidence interval (CI) for CR-BSI incidence rates. Odds ratio (OR) and 95% CI were used to identify risk factors associated with the CR-BSI by using logistic regression models. Model building and selection for CR-BSI were based on Bayesian Model Average. Goodness-of-fit of the models was accessed by Pearson chi-square and deviance statistic. We used 1000 bootstrap replicates to determine more important variables in the final model.

## Results

### Results of training on BSI preventive practices for the 71 nurses of DON and the auditing and feedback of aseptic technique

The proportion of nurses with adequate knowledge was 92% (65) after the training compared with 30% (21) before the training. In the practical section before the training, 28% (20) of the nurses did not comply with handwashing and aseptic technique, 21% (15) had faults with skin disinfection, 17% (12) touched the syringes’ needles during withdrawing liquid medicines, and 13% (9) used aseptic dressings for the catheter inadequately. After the training, the 65 nurses adhered to handwashing and aseptic technique during the insertion of intravenous catheters and maximal sterile barrier precautions. About 8% (6) were re-trained on the BSI preventive practices before obtaining adequate knowledge.

During the intervention period, 4147 peripheral intravenous catheters were observed. All the catheters were located in appropriate positions, and 96% (3998) were cared for and maintained appropriately at catheter sites. Of the 4% (149 catheters with inappropriate care and maintenance), 2% (66) were due to oedema at catheter sites, 1.5% (61) used inappropriate catheter dressings, and 0.5% (22) did not remove unused intravenous catheters. Among 300 umbilical catheters observed, 3% (9) were placed for more than 14 days.

### Characteristics of neonates in the pre- and post-intervention periods

Among the 2551 neonates with intravenous catheter insertion admitted to the DON in the study period, 2225 met inclusion criteria, of which 1027 and 1198 were enrolled in the pre- and post-intervention periods, respectively. The difference of characteristics of gestational age, gender, weight at birth, premature birth, neonates with congenital diseases, endotracheal intubation, ventilator days, blood infusion, reasons for intravenous catheter insertion of blood infusion and intravenous injection, episodes of HAI, duration of hospitalisation, and status of discharge among neonates between pre-and post-intervention periods were significant (*p* < 0.05). There were no significant differences of days of age at catheter insertion and total parenteral nutrition among neonates between the two periods (Table 2).

**Table 2:** Characteristics of neonates with intravenous catheter insertion for ≥48 h in pre- and post-intervention periods in Hung Vuong Hospital, Vietnam

Characteristics	Pre-intervention neonate (*n* = 1027)	Post-intervention neonate (*n* = 1198)	Total (*n* = 2225)	*p*-value
Gestational age (mean, SD)	33.6 ± 3.6	34.7 ± 3.7	34.3 ± 3.7	< 0.001^a^
Gender (n, %)				0.04^b^
Male	549 (53.5)	694 (57.9)	1243 (55.9)	
Female	478 (46.5)	504 (42.1)	982 (44.1)	
Days of age with catheter insertion (median, range)	1 (1, 27)	1 (1, 122)	1 (1, 122)	0.45^c^
Weight at birth (gram, mean, SD)	2135 ± 755	2276 ± 780	2211 ± 771	< 0.001^a^
Methods of delivery (n, %)				0.14^b^
Vaginal	568 (55.3)	624 (52.1)	1192 (53.6)	
Cesarean section	459 (44.7)	574 (47.9)	1033 (46.4)	
Premature (< 37 weeks, (n, %))	699 (68.1)	738 (61.6)	1437 (64.6)	< 0.01^b^
Neonates with congenital diseases (n, %)	261 (25.4)	218 (18.2)	479 (21.5)	< 0.001^b^
Endotracheal intubation (n, %)	208 (20.3)	179 (14.9)	387 (17.4)	< 0.01^b^
Ventilator days (median, IQR)	1.5 (1.0, 3.0)	2.0 (1.0, 4.5)	2.0 (1.0, 3.5)	< 0.001^c^
Reasons for intravenous catheter insertion (n, %)				
Blood infusion	157 (15.3)	133 (11.1)	290 (13.0)	< 0.01^b^
Total parenteral nutrition	1017 (99.0)	1177 (98.2)	2194 (98.6)	0.17^d^
Intravenous injection	783 (76.2)	827 (69.0)	1610 (72.4)	< 0.001^b^
Episodes of hospital-acquired infection (mean, SD)	1.32 ± 0.59	0.17 ± 0.46	0.39 ± 0.66	< 0.001^a^
Days of hospitalization (mean, SD)	15.5 ± 19.2	12.5 ± 15.1	13.9 ± 17.2	< 0.001^a^
Status of discharge (n, %)				< 0.01^b^
Death	113 (11.0)	80 (6.7)	193 (8.7)	
Cured	874 (85.1)	1070 (89.3)	1944 (87.4)	
Hospitalization	40 (3.9)	48 (4.0)	88 (4.0)	

*SD* standard deviation, *IQR* interquartile range

^a^t-test; ^b^Chi-square test; ^c^Mann Whitney U test, ^d^Fisher’s exact test

During the study period, 16,723 catheter-days were included in the final analysis, of which 8324 were included in the post-intervention period. After the intervention, the CR-BSI rate significantly decreased from 6.31 infections per 1000 catheter-days in the pre-intervention period to 3.84 in the post-intervention period (RR = 0.61, 95% CI 0.39–0.94, Table 3). The characteristics showed in Table 2 were used for model building and selection; however, no potential confounding factors were found in multivariate Poisson regression (the effects changed less than 10%).

**Table 3:** Catheter-related bloodstream infection rate in pre- and post-intervention periods in Hung Vuong Hospital, Vietnam

Period	Cases	Catheter-days	Rate/1000 catheter-days	RR (95% CI)
Pre-intervention	53	8399	6.31	
Post-intervention	32	8324	3.84	0.61 (0.39–0.94)

*RR* rate ratio, *CI* confidence interval Wald χ2 statistics were used to test the significance of individual coefficients

### Characteristics of catheter-related bloodstream infection cases in the pre- and post-intervention periods

During the study period, we identified a total of 85 CR-BSI cases, of which 53 occurred during the pre-intervention period and 32 occurred in the post-intervention period. Approximately 44% (37) of CR-BSI cases were diagnosed by clinical definition, 29% (25) by laboratory-confirmed definition, and 27% (23) by clinical and common skin contaminant definition. Among the symptoms of the 85 cases, 8% were febrile, 44% had apnoea, and 12% had bradycardia, but there were no significant differences among the symptoms of cases between the pre- and post-intervention periods (Table 4).

**Table 4:** Characteristics of neonates with catheter-related bloodstream infection in pre- and post-intervention periods in Hung Vuong Hospital, Vietnam

Characteristics	Pre-intervention cases (*n* = 53)	Post-intervention cases (*n* = 32)	Total (*n* = 85)
Types of cases diagnosed blood stream infections by case definitions (BSI, n, %)			
Laboratory-confirmed BSI	20 (37.7)	5 (15.6)	25 (29.4)
Clinical and common skin contaminant BSI	13 (24.5)	10 (31.2)	23 (27.1)
Clinical BSI	20 (37.7)	17 (53.1)	37 (43.5)
Fever > 38 °C (n, %)	5 (9.4)	2 (6.2)	7 (8.2)
Apnea (n, %)	21 (39.6)	16 (50.0)	37 (43.5)
Bradycardia (n, %)	7 (13.2)	3 (9.4)	10 (11.8)
Positive blood culture (n, %)	33 (62.3)	15 (55.6)	48 (60.0)
Coagulase*-*negative staphylococci	*10 (30.3)*	*2 (13.3)*	*12 (25.0)*
*Enterobacter (aerogenes, gergoviae,* spp.)	*10 (30.3)*	*8 (53.3)*	*18 (37.5)*
*Escherichia coli*	*10 (30.3)*	*3 (20.0)*	*13 (27.1)*
*Acinetobacter* spp.	*2 (6.1)*	*0 (0.0)*	*2 (4.2)*
*Escherichia vulneris*	*1 (3.0)*	*1 (6.7)*	*2 (4.2)*
*Stenotrophomonas maltophilia*	*0 (0.0)*	*1 (6.7)*	*1 (2.1)*
Episodes of hospital-acquired infection (mean, SD)	1.47 ± 0.67	1.19 ± 0.59	1.36 ± 0.65
Status of discharge of cases (n, %)			
Death	20 (37.7)	11 (34.4)	31 (36.5)
Cured	30 (56.6)	20 (62.5)	50 (58.8)
Transferred	3 (5.7)	1 (3.1)	4 (4.7)
Days from intravenous catheter insertion to diagnosis (mean, SD)	7.11 ± 5.45	6.34 ± 3.36	6.82 ± 4.76
Days of hospitalization (mean, SD)	33.9 ± 39.2	30.3 ± 18.8	32.5 ± 32.9

*SD* standard deviation

Among blood samples taken from the CR-BSI cases, 48 were positive, of which 33 and 15 samples were positive in the pre- and post-intervention periods, respectively. The most common causes in the pre-intervention period were *Enterobacter* spp. (30%), *Escherichia coli* (30%), and coagulase-negative staphylococci (30%), while causes in post-intervention period were *Enterobacter* spp. (53%) and *Escherichia coli* (20%). Two cases (one in each of the stages) were positive for *Escherichia vulneris*; both received TPN via a catheter and were premature with gestational age at birth of 32 and 26 weeks and weight at birth of 1270 g and 900 g, respectively. Six MDR *Enterobacter* spp. cases that were resistant to amoxicillin and clavulanate, ceftazidime, and ceftriaxone were confirmed. Episodes of HAI among CR-BSI cases in the post-intervention period decreased (1.19 ± 0.59 vs. 1.47 ± 0.67 in the pre-intervention period). The proportion of dead CR-BSI cases in the post-intervention period was similar to that of the pre-intervention period. The mean of the number of days from catheter insertion to diagnosis and the mean of the number of days of hospitalisation in the post-intervention period were shorter than those in the pre-intervention period (Table 4).

### Risk factors associated with catheter-related bloodstream infection cases in the pre- and post-intervention periods

Among potential risk factors, preterm birth, hospital stays, duration of intravenous catheters, use of intravenous injection, use of endotracheal intubation, and use of umbilical catheter were associated with the CR-BSIs in univariate analysis. When applying logistic regression models in multivariate analysis, only days of intravenous catheter (OR = 1.05, 95% CI 1.03–1.08), use of endotracheal intubation (OR = 2.27, 95% CI 1.27–4.06), and intravenous injection (OR = 8.50, 95% CI 1.14–63.4) were still significant (Table 5). Results of the bootstrap replicates of the final model showed that the duration of the intravenous catheters was the most important variable, while the intravenous injection had the least effect in the final model.

**Table 5:** Univariate and adjusted odds ratios for the association between risk factors and catheter-related bloodstream infection in Hung Vuong Hospital, Vietnam

Risk factors	Cases (*n* = 85)	Non-cases (*n* = 2140)	OR (95% CI)	Adjusted OR (95% CI)
Gestational age (weeks)	30.4 ± 3.10	34.5 ± 3.62	0.75 (0.70–0.80)	0.83 (0.75–0.92)
Gender (n, %)			0.98 (0.63–1.51)	–
*Male*	47 (55.3)	1196 (55.9)		
*Female*	38 (44.7)	944 (44.1)		
Weight at birth (gram)	1451 ± 468	2242 ± 766	0.98 (0.63–1.51)	–
Preterm birth (n, %)	78 (91.8)	1359 (63.5)	6.40 (2.94–13.95)	0.93 (0.27–3.18)
Congenital diseases (n, %)	22 (25.9)	457 (21.4)	1.29 (0.78–2.11)	–
Methods of delivery (n, %)				
*Cesarean section*	28 (32.9)	1005 (47.0)	0.55 (0.35–0.88)	0.96 (0.54–1.71)
*Vaginal*	57 (67.1)	1135 (53.0)		
Days of hospital stays	33.0 ± 33.2	13.2 ± 15.8	2.79 (2.78–2.81)	1.01 (0.99–1.02)
Days of intravenous catheter	19.7 ± 11.0	7.03 ± 7.06	3.03 (2.96–3.09)	1.05 (1.03–1.08)
Intravenous injection (n, %)	83 (97.6)	1527 (71.4)	16.7 (4.09–67.9)	8.50 (1.14–63.4)
Total parenteral nutrition (n, %)	85 (100)	2109 (98.6)	–	
Use of endotracheal intubation (n, %)	49 (57.6)	338 (15.8)	7.26 (4.65–11.3)	2.27 (1.27–4.06)
Days of ventilator	5.98 ± 6.67	3.71 ± 7.30	1.03 (0.99–1.07)	–
Use of umbilical intravenous catheter	23 (27.1)	119 (5.6)	6.30 (3.77–10.5)	1.63 (0.87–3.04)
Days of using umbilical intravenous infusion	8.13 ± 5.48	5.73 ± 4.31	1.10 (0.99–1.22)	–

*OR* odds ratio, *CI* confidence interval

Wald χ2 statistics were used to test the significance of individual coefficients

## Discussion

This study demonstrated that implementation of basic infection control interventions significantly reduced CR-BSIs in NICU of a tertiary maternity hospital. The post-intervention incidence rate was much lower than that of a study conducted at the HVH in 2007 (15.3 per 1000 catheter-days) and of a study conducted at Children’s Hospital No. 1, Vietnam in 2005 (7.5 per 1000 catheter-days) [12, 13]. Compared with neonatal care units in other countries, the post-intervention incidence rate in this study was also lower than the rates of studies conducted in the Philippines during 2005–2009 and in Turkey in 2008 [4, 17]. However, the rate after the intervention in our study was still higher than that of studies conducted in the developed countries, e.g. in the United States and Spain [18–21]. Although there were differences of the characteristics of neonates and catheter insertions in these studies (e.g. most neonates in our study were inserted by peripheral intravenous catheters and in a few cases with umbilical catheters), the rates of our study indicated that the intervention was effective, especially in resource-poor settings in Vietnam.

The effective intervention must have resulted from significant improvements in the nurses’ knowledge and compliance with the CR-BSI preventive practices after the training. In a hand-hygiene–focused intervention in Brazil (2009), the rate of CR-BSI significantly decreased from 24.1 to 14.9 per 1000 catheter-days [22]. Several studies have shown that intensified training and educational programmes on hand hygiene and aseptic technique decreased the risk of CR-BSIs [20, 21, 23–25]. Our study not only implemented education interventions, but also included other interventions, such as auditing and feedback of compliance with aseptic technique and reorganisation of TPN preparation by preparing it under a laminar airflow hood in a clean room in DOP as recommended by WHO. The intervention probably led to a significant decrease of episodes of HAI among CR-BSI cases as well as hospital stays, number of deaths, and episodes of HAI among neonates with intravenous catheter insertions in the post-intervention period.

Most positive blood culture bacteria in our study were major common nosocomial pathogens that were consistent with other studies [8, 26, 27]. The decrease of coagulase-negative staphylococci, *Enterobacter* spp., *Escherichia coli*, and *Acinetobacter spp.* detected in blood samples in the post-intervention period highlights the effectiveness of BSI preventive practices, including auditing of and feedback about aseptic technique during care and maintenance of catheters [28]. Because of limited resources, we could not evaluate the effectiveness of each individual intervention. However, the types of interventions in our study may be appropriate for similar studies conducted in neonatal intensive care units in resource-poor settings.

*Escherichia vulneris* is usually found in wounds. It is an opportunistic pathogen and can cause sepsis followed by complicated diarrhoea [29]. *Escherichia vulneris* has rarely been reported as a CR-BSI pathogen. To our knowledge, only one CR-BSI case caused by *Escherichia vulneris* has been reported in the literature: the case-patient had poor nutritional status and received TPN via a central catheter [30]. In our study, we detected two cases, both premature neonates who received TPN via a catheter, that were positive for *Escherichia vulneris*. Hence, the pathogen should be suspected in premature neonates receiving TPN via a catheter. *Enterobacter* spp. was a common CR-BSI pathogen found on nurses’ hands and MRDO [10, 31, 32]; however, the percentage of MDR *Enterobacter* spp. among reported CR-BSI pathogens was relatively high (13%) compared with previous studies, and no CR-BSI MDR *Enterobacter* spp. was found in intensive care units in developing countries [31–33].

TPN is a controversial risk factor in the literature [34]. TPN was a critical risk factor of CR-BSI [9, 11], but it was not found to be a risk factor in our study, which is consistent with other studies [35, 36]. This could be due to our intervention of relocating TPN preparation from DON to a clean room in DOP and preparing parenteral nutrition under a laminar airflow hood. Birth weight and preterm neonates have not been identified as risk factors in this study or in other studies [9, 35]. Instead, we identified risk factors that included the days of intravenous catheters, intravenous injection, and the use of endotracheal intubation that were consistent with other studies [19, 25, 35–37]. Although the interventions in the study were shown to be effective, three risk factors are related directly to catheter usage. It is clear that catheter care and other BSI preventive practices should be regularly monitored and good practices should be maintained. The duration and types of catheters used for neonates need to be further studied.

## Conclusions

The basic infection control interventions in this study had a major effect in reducing the incidence of CR-BSI in resource-poor settings. The BSI preventive practices, including auditing of and feedback to nurses, should be maintained, especially removing unnecessary intravenous catheters and stringently indicating the use of intravenous injection and endotracheal intubation to reduce the risk of CR-BSI.

## Data Availability

Available.

## Abbreviations

BSI: Bloodstream infection
CDC: Centers for Disease Control and Prevention
CI: Confidence interval
CR-BSI: Catheter related bloodstream infections
DON: Department of Neonatal
DOP: Department of Pharmaceuticals
HAI: Hospital-acquired infections
HVH: Hung Vuong Hospital
MDR: Multi-drug–resistant
MDRO: Multi-drug–resistant organisms
NICU: Neonatal intensive care unit
OR: Odds ratio
RR: Rate ratio
TPN: Total parenteral nutrition
WHO: World Health Organization
